# Antiviral Activity of Baicalein and Quercetin against the Japanese Encephalitis Virus

**DOI:** 10.3390/ijms131216785

**Published:** 2012-12-07

**Authors:** Jefree Johari, Aynaz Kianmehr, Mohd Rais Mustafa, Sazaly Abubakar, Keivan Zandi

**Affiliations:** 1 Tropical Infectious Disease Research and Education Center (TIDREC), Department of Medical Microbiology, Faculty of Medicine, University of Malaya, 50603 Kuala Lumpur, Malaysia; E-Mails: jefree.johari@um.edu.my (J.J.); aynaz99@yahoo.com (A.K.); sazaly@um.edu.my (S.A.); 2 Department of Pharmacology, Faculty of Medicine, University of Malaya, 50603 Kuala Lumpur, Malaysia; E-Mail: rais@um.edu.my

**Keywords:** antiviral, Japanese encephalitis virus, infectious disease, flavonoid, baicalein, quercetin

## Abstract

Japanese encephalitis (JE), a mosquito-borne viral disease, is endemic to the entire east and southeast Asia, and some other parts of the world. Currently, there is no effective therapeutic available for JE; therefore, finding the effective antiviral agent against JEV replication is crucial. In the present study, the *in vitro* antiviral activity of baicalein and quercetin, two purportedly antiviral bioflavonoids, was evaluated against Japanese encephalitis virus (JEV) replication in Vero cells. Anti-JEV activities of these compounds were examined on different stages of JEV replication cycle. The effects of the compounds on virus replication were determined by foci forming unit reduction assay (FFURA) and quantitative RT-PCR. Baicalein showed potent antiviral activity with IC_50_ = 14.28 μg/mL when it was introduced to the Vero cells after adsorption of JEV. Quercetin exhibited weak anti-JEV effects with IC_50_ = 212.1 μg/mL when the JEV infected cells were treated with the compound after virus adsorption. However, baicalein exhibited significant effect against JEV adsorption with IC_50_ = 7.27 μg/mL while quercetin did not show any anti-adsorption activity. Baicalein also exhibited direct extracellular virucidal activity on JEV with IC_50_ = 3.44 μg/mL. However, results of quantitative RT-PCR experiments confirmed the findings from FFURA. This study demonstrated that baicalein should be considered as an appropriate candidate for further investigations, such as the study of molecular and cellular mechanism(s) of action and *in vivo* evaluation for the development of an effective antiviral compound against Japanese encephalitis virus.

## 1. Introduction

The Japanese encephalitis virus (JEV) is an arthropod-borne virus emerging from the *Flaviviridae* family. It is one of the most important causative agents for viral encephalitis in human that can cause symptoms ranging from febrile to mortal illnesses notably in children with 30,000–50,000 cases around the world annually. JEV infection is endemic in eastern and southern Asia including in Nepal, Indonesia, China, Thailand, Australia, Sapian Island, Pakistan and the Torres Strait [1–4]. The fatality rate of JEV infection is estimated at about 30%, with life-long neurological impairments and sequels among half of the survivors [5]. Japanese encephalitis virus is an enveloped virus with a positive sense single stranded RNA of 11 kb in length. Its genome formed a single long open reading frame (ORF) flanked by the 5′-UTR and 3′-UTR. The ORF is translated into a polyprotein, which is processed by viral and cellular proteases to yield three structural proteins called capsid (C), pre-membrane (prM), envelope (E), and seven non-structural (NS) proteins namely NS1, NS2 A/B, NS3, NS4 A/B and NS5 [6]. There are still concerns of efficacy, long term safety and cost for all the current available vaccines resulting in a high percentage of unvaccinated population in endemic regions, which translates as many cases of JE in these regions [7]. Thus, there is still a need to find effective antiviral against JEV [8]. There have been efforts to find effective antiviral substance among the natural compounds such as plant or algal derived compounds [9,10]. Recently, *in vitro* anti-JEV activities of two different types of bioflavonoids namely kaempferol and daidzin were evaluated and it was shown that kaempferol is more effective against JEV replication compared to daidzin [11]. Compounds from natural resources are regarded as possible alternatives as they may show low side effects and easily accessible from nature. Among the natural compounds, flavonoids driven from fruits, roots, nuts, seeds, bark, steams and flowers of plants have been investigated to display numerous possible biological benefits [12–14]. Baicalein a flavonoid among the flavones subgroup has exhibited antiviral activities against a number of viruses including herpes viruses, some human adenoviruses and respiratory syncytial virus [15,16]. In addition, quercetin a flavonoid from flavonol subgroup has showed antiviral activities against viruses such as influenza virus, some herpes viruses, porcine epidemic diarrhea virus and some types of human adenoviruses [15–20]. We have recently reported the inhibitory effect of quercetin against *in vitro* replication of dengue virus [21]. Here we evaluate the *in vitro* antiviral activity of baicalein and quercetin against different stages of JEV replication cycle.

## 2. Results

### 2.1. Cytotoxicity of Bioflavonoids

A MTT(3-(4,5-dimethylthiazol-2-yl)-2,5-diphenyltetrazolium bromide) assay was used to determine the cytotoxicity effect of baicalein and quercetin on Vero cells in which the half maximal cytotoxic concentration (CC_50_) value of each compound was calculated. Vero cells were treated by bioflavonoids for two days, which was the same duration as in the antiviral activity assay. Results illustrate a higher cytotoxic value of CC_50_ = 115.2 ± 0.2 μg/mL for baicalein compared to quercetin with CC_50_ = 256.5 ± 0.17 μg/mL. Treated cells with vehicle control, 1% DMSO did not show any cytotoxicity against Vero cells.

### 2.2. Antiviral Activity of Baicalein and Quercetin against JEV

Baicalein and quercetin were examined for the potential antiviral effects, (i) for their prophylactic activity, (ii) for their intracellular antiviral activity after virus adsorption, (iii) against virus adsorption to the cells and (iv) adding directly to the virus suspension in order to examine the compounds’ direct virucidal effect.

**Figure 1:**
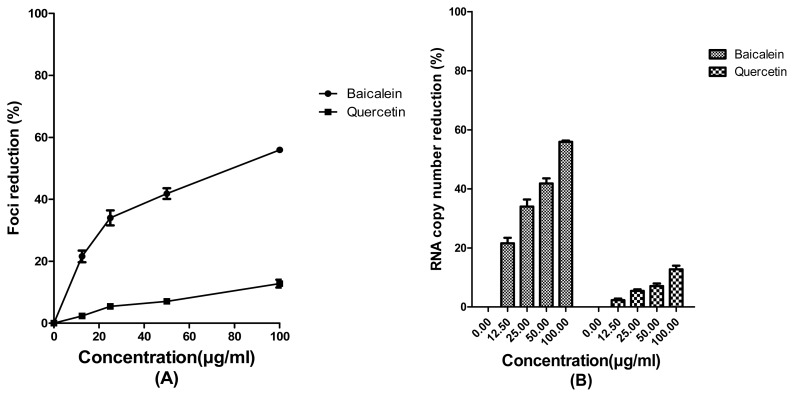
Evaluation of the prophylactic effects of baicalein and quercetin on Japanese encephalitis virus (JEV) *in vitro* replication. Foci forming unit reduction assay was used to evaluate the prophylactic effects (**A**) and the respective JEV RNA copies were quantified using qRT-PCR (**B**). All experiments were performed in triplicates. Data were plotted using Graph Pad Prism Version 5 (Graph Pad Software Inc., San Diego, CA, USA, 2005).

Results of prophylactic treatment with the tested bioflavonoids showed that 25 μg/mL of baicalein and quercetin could decrease the copy number of JEV RNA 34% ± 2.3% and 5.45% ± 0.54% respectively when compared to the non-treated cells (Figure 1B). The half maximal concentration (IC_50_) value for baicalein based on the data from FFURA was 84.18 ± 1.08 μg/mL (Figure 1A). Quercetin did not exhibit a significant prophylactic activity, therefore its IC_50_ value was not considered.

**Figure 2:**
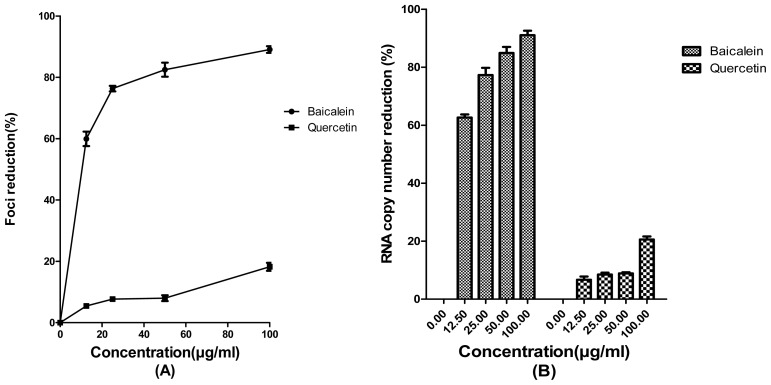
Effects of baicalein and quercetin against JEV adsorption to the Vero cells. Foci forming unit reduction assay was used to evaluate the antiviral activities (**A**) and the respective JEV RNA copies were quantified using qRT-PCR (**B**); All experiments were performed in triplicates. Data were plotted using Graph Pad Prism Version 5.

Indeed, baicalein showed anti-adsorption activity against JEV with IC_50_ = 7.27 ± 1.08 μg/mL (Figure 2A). In addition, JEV RNA copy number decreased 77.3% ± 2.4% in the presence of 25 μg/mL of baicalein during the viral adsorption period (Figure 2B). There was no significant anti-adsorption activity against JEV by quercetin and the JEV copy number decreased 8.46% ± 0.64% in the presence of 25 μg/mL of that compound (Figure 2B).

**Figure 3:**
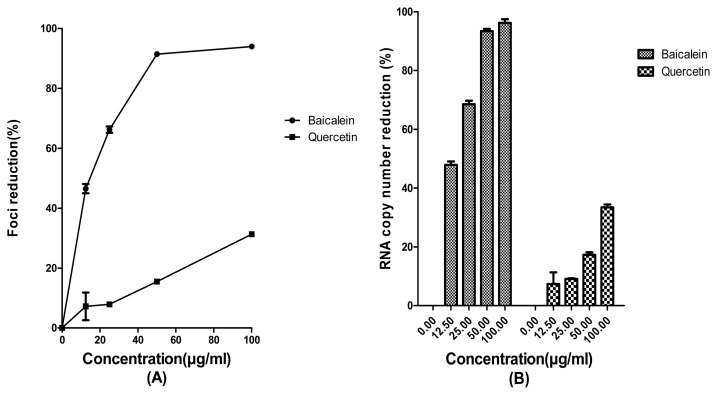
Antiviral activity of baicalein and quercetin against JEV intracellular replication. Foci forming unit reduction assay was used to evaluate the antiviral activities (**A**) and the respective JEV RNA copies were quantified using qRT-PCR (**B**). All experiments were performed in triplicates. Data were plotted using Graph Pad Prism Version 5.

In post adsorption assay, baicalein exhibited potent antiviral activity against JEV with IC_50_ = 5.8 ± 1.09 μg/mL (Figure 3A). The copy number of viral RNA was decreased 68.46% ± 1.19% when the infected cells were treated with 25 μg/mL (Figure 3B). The IC_50_ for quercetin at this phase of experiment was 212.1 ± 0.9 μg/mL but it is too closed to the CC_50_ value for quercetin. The copy number of JEV RNA decreased 9.1% ± 0.2% in presence of 50 μg/mL of that bioflavonoid (Figure 3B).

**Figure 4:**
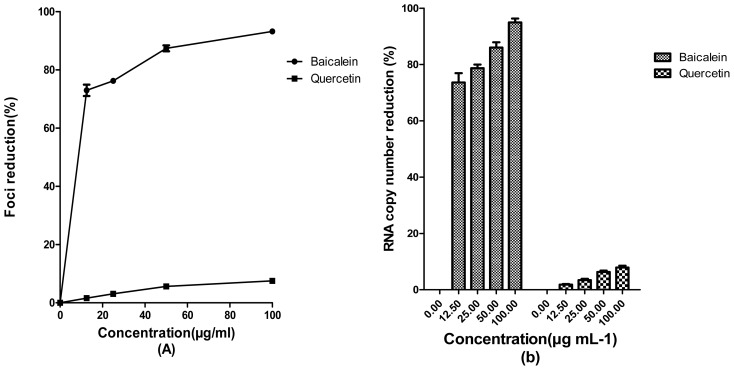
Direct virucidal activity of baicalein and quercetin against JEV. Foci forming unit reduction assay was used to determine the direct virucidal activities of the compounds against extracellular JEV (**A**) and the respective JEV RNA copies were quantified using qRT-PCR (**B**). All experiments were performed in triplicates. Data were plotted using Graph Pad Prism Version 5.

Baicalein showed direct virucidal activity with IC_50_ = 3.44 ± 1.04 μg/mL (Figure 4A) against JEV particles. However, qRT-PCR analysis showed that 25 μg/mL of baicalein decreased the DENV-2 RNA production 78.7% ± 1.2% (Figure 4B). Meanwhile, quercetin did not exhibit any significant direct extracellular activity JEV (Figure 4A) and 25 μg/mL of that bioflavonoid could decrease the JEV RNA copy number only for 3.6% ± 0.5% (Figure 4B).

## 3. Discussion

Bioflavonoids are phenolic compounds that are generally found in plants. Several investigations have shown their beneficial effects on human health [22,23]. Numerous studies have shown that some flavonoids exhibited antiviral effects against a number of viruses including dengue virus, hepatitis B virus, human cytomegalovirus and few others [15–18,21]. In one study, it was demonstrated that baicalein was the most effective antiviral against cytomegalovirus compared to the other tested flavonoids such as quercetin, apigenin, naringenin, daidzein, luteolin and some other flavonoids [15]. Indeed, it was demonstrated that baicalein increased the *in vitro* and *in vivo* antiviral activity of ribavirin against influenza virus significantly [24]. Recently, through theantiviral activity of kaempferol and daidzin, two types of flavonoids against Japanese encephalitis virus were reported [11]. In the present study we also have demonstrated that baicalein exhibited potent *in vitro* anti-JEV effects at all different stages of JEV infection compared to the quercetin. The selectivity indices (SI) for baicalein for all stages were significantly higher than that of quercetin. Baicalein exhibited significant direct virucidal activity (SI = 33.4) as well as intracellular anti-JEV activity (SI = 15.8) and anti-adsorption activity (SI = 15.8) but its prophylactic activity was not significant (SI = 1.3). On the other hand, quercetin exhibited anti-JEV activity only when used against JEV adsorption to the cells (SI = 1.2). It was also demonstrated that the production of JEV-RNA decreased more than 17% in the presence of 50 μg/mL of quercetin when it was used after virus adsorption (Figure 3B). Similar to our previous findings with the anti-dengue activity of quercetin [21] there was no significant anti-JEV effect when it was tested for its prophylactic, anti-adsorption or its direct virucidal activity against JEV.

The mechanism(s) conferring the antiviral properties of baicalein against JEV is unknown. Regarding the potent virucidal activity of baicalein against extracellular JEV particles with SI = 33.4 might be one of the main mechanisms that can explain anti-JEV activity of baicalein. A possible mechanism, which might explain the prophylactic activity of baicalein against JEV replication, is about the accumulation of the compound in the cells during the treatment. However, prevention of virus adsorption to the cells is another possibility that might lead to the inhibition of virus entry to the cells.

Another possibility for mechanism of action for intracellular anti-JEV activity is binding of baicalein to the viral RNA. This possibility was shown in a recent study for two other flavonoids, kaempferol and daidzin using the JEV frame shift RNA [11]. Results from qRT-PCR indicate that significant reduction of JEV RNA copy number is consistent with inhibition of JEV replication and/or RNA synthesis together with transcription.

The other possible mechanism of action for baicalein includes interaction with JEV structural and/or non-structural protein(s). It has previously been shown that baicalein affects Sendai virus replication through inhibition of virus hemagglutinin-neuraminidase activity [13]. Other studies have also shown that baicalein binds to HIV-1 integrase and reverse transcriptase enzymes [25,26]. These findings suggest that baicalein can bind to a number of viral enzymes important for their replication.

On the other hand, quercetin a flavonoid with reported antiviral activity against hepatitis C virus (HCV), through binding and inactivating viral NS3 protease [27] did not show a significant anti-JEV activity in our study. In contrast with baicalein, which affected JEV adsorption and intracellular replication, quercetin showed no similar effects. Moreover, baicalein showed potent virucidal activity against extracellular viruses where quercetin did not exhibit the same effect. These findings are similar to our earlier experiments and findings on the effects of quercetin against dengue virus adsorption to the cells [21]. Quercetin might be considered as an anti-JEV compound upon further modifications to improve its anti-JEV effects. This possibility for influenza virus has been shown in one study that isoquercetin, a derivative of quercetin is more effective against influenza virus compared to quercetin [20].

In all our experiments, we showed that 0.5% of DMSO, the solvent used for our tested bioflavonoids did not exhibit any antiviral activity against JEV and this rule out any probable antiviral activity from DMSO.

## 4. Experimental Section

### 4.1. Bioflavonoids

Two types of bioflavonoid, quercetin (Sigma-Aldrich, St. Louis, MO, USA) and baicalein (Indofine Chemical Co. Inc., Hillsborough, NJ, USA) were evaluated for their potential antiviral activity against Japanese encephalitis virus. Dimethyl sulfoxide (DMSO) (Sigma-Aldrich, St. Louis, MO, USA) was used as a proper solvent to prepare the compounds’ stock solution (20 mg/mL). The prepared stock solutions were stored at −20 °C. Stock solution was diluted using cell culture medium and sterilized by syringe filter with 0.2 micron pore size (Millipore, Billerica, MA, USA).

### 4.2. Cell and Virus

Vero cell line derived from the kidney of African green monkey was used in this study. The cell line was maintained and propagated in Eagle’s Minimum Essential Medium (EMEM) (Gibco, New York, NY, USA) containing 10% fetal bovine serum (Gibco, New York, NY, USA). Cultured cells were incubated at 37 °C in 5% CO_2_ humidified chamber. At the time of virus propagation and antiviral experiments, the serum concentration was reduced to 2%. Japanese encephalitis virus (JEV) (Accession Number: HE861351) was propagated and harvested after cytopathic effects (CPE) presentation four days post-infection. Viral stock was titred using foci forming assay (FFA) and stored at −70 °C.

### 4.3. *In Vitro* Cytotoxicity Assay

Cytotoxicity assays of baicalein and quercetin against Vero cells were performed using the MTT assay method. Briefly, a confluent monolayer of Vero cell line was prepared in 96-well microplate and treated by different concentrations of each compound in triplicates. The treated cells were incubated for two days at 37 °C that is consistent with the incubation period for anti-JEV activity assay. After two days treatment, the MTT assay was performed strictly according to the manufacturer’s recommendation and as described earlier [14]. Dose-response curve was plotted using Graph Pad Prism 5 (Graph Pad Software Inc., San Diego, CA, USA, 2005) and the half maximal cytotoxic concentration (CC_50_) was determined from the plot.

### 4.4. Foci Forming Reduction Assay (FFURA)

Antiviral activities of baicalein and quercetin were evaluated by measuring the reduction in the number of viral foci, which was formed following treatments. Briefly, confluent monolayers of Vero cells were prepared in 24-well cell culture microplate. Infected cell monolyaers were treated using different procedures that will be described later and overlaid with 1.5% CMC containing EMEM with 2% FBS and incubated at 37 °C in 5% CO_2_ humidified chamber for two days. Viral foci formed were stained using JEV monoclonal antibody (Pierce, Rockford, IL, USA) and goat anti-rabbit IgG conjugated with horse-radish peroxidase (HRP). Foci were counted under a stereomicroscope and expressed as Foci-Forming-Unit (FFU).

Reduction in number of viral foci (RF%) compared against the mock treated controls was calculated as follows: RF (%) = (*C* − *T*) × 100/*C*

Where, *C* is the mean of the number of foci for the mock treated control well infected with JEV and *T* is the mean of the number of foci formed in the JEV infected cell cultures.

### 4.5. Assays of Antiviral Activity

Antiviral assays were performed as described previously [21]. The prophylactic effects of the compounds on JEV replication was evaluated by adding the different concentrations of each compound separately to the Vero monolayer cells in triplicates, 5 h prior to JEV infection. Cells were then washed using sterile PBS to remove the flavonoids and infected with JEV to give an estimated infection of 200 FFU (0.01 MOI) per well and kept at 37 °C for 1 h. The cells were then washed with PBS to eliminate unabsorbed viruses, overlaid by 1.5 CMC containing EMEM with 2% and incubated for another two days.

The antiviral activity of the compounds against intracellular JEV replication was evaluated by inoculating of 200 FFU of virus (0.01 MOI) to each well in triplicates. After 1 h incubation at 37 °C for virus adsorption, the cells were washed with PBS and different concentrations of each compound that prepared in 1.5% CMC containing EMEM with 2% FBS were added to the cells, followed by two days of incubation at 37 °C.

To determine the effect of bioflavonoids against adsorption of JEV to the host cells, Vero cells at 80% confluence were infected with 200 FFU of JEV in the presence or absence of different concentrations of each compound. After washing, the infected cells were overlaid by 1.5% CMC containing EMEM with 2% FBS and incubated at 37 °C for two days.

### 4.6. Direct Virucidal Activity Assay

Extracellular effects of the baicalein and quercetin against JEV particles were investigated by incubating the JEV suspension containing 10^5^ FFU (5 MOI) with an equal volume of the different concentrations of each compound for 2 h at 37 °C. Then, Vero cells were infected with the 1000 fold diluted treated viral suspension in triplicates. After 1 h adsorption at 37 °C, cells were washed twice with PBS in order to remove unattached viruses. Cells were overlaid by 1.5% CMC containing EMEM with 2% FBS and incubated at 37 °C for two days.

### 4.7. Quantitative RT-PCR (qRT-PCR)

Quantitative RT-PCR was performed to evaluate the effects of baicalein and quercetin on JEV replication by quantifying the JEV RNA copy number based on a method described previously with some modifications [25]. Briefly, intracellular JEV RNAs were harvested from the JEV-infected Vero cells. Viral RNA was extracted using RNA extraction kits (Qiagen, Hilden, Germany). Quantitative RT-PCR assay was performed using the SensiMix SYBR green reagent (Quantace, Watford, UK) in a reaction mix consisting of 7.4 μL of ddH2O, 10 μL of 2× SensiMix One-Step, 0.4 μL of 50× SYBR Green solution, 10 units of RNAse Inhibitor, 50 pmol of forward 5′-AGAGCGGGGAAAAAGGTCAT-3′ and reverse 5′-CTTCACGCTCTTCCTACAGT-3′ JEV amplification primers [28,29]. All samples were assayed in triplicates. The amplifications were done on the StepOnePlus™ Real-Time PCR System (Applied Biosystems, Foster City, CA, USA) with the following thermal conditions: reverse transcription at 50 °C for 30 min, initial denaturation at 95 °C for 10 min, followed by 40 cycles of 95 °C for 5 s and 60 °C for 10 s. Melting curve analysis was subsequently performed at temperature from 60 °C to 98 °C to verify the assay specificity. For absolute quantitation of the viral RNA, a standard curve was established with a serially diluted *in vitro* transcribed RNA of JEV with known copy number.

### 4.8. Statistical Analysis

Graph Pad Prism for Windows, version 5 (Graph Pad Software Inc., San Diego, CA, USA, 2005) was used to calculate half maximal cytotoxic concentration (CC_50_). The same software was used to calculate half maximal inhibitory concentration (IC_50_) values of the tested compounds by analyzing the data through the non-linear regression analysis. Selectivity Index value (SI) was determined as the ratio of CC_50_ to IC_50_ for each compound.

## 5. Conclusions

In summary, our study demonstrates that baicalein as a flavonoid possesses a series of significant antiviral activities against the different stages of *in vitro* JEV replication. Baicalein should be considered for further investigations as a possible novel antivirus against JEV.
